# Tris(ethyl­enedi­amine)­cobalt(II) dichloride

**DOI:** 10.1107/S1600536813013135

**Published:** 2013-05-18

**Authors:** Kristin Cooke, Andrei V. Olenev, Kirill Kovnir

**Affiliations:** a Department of Chemistry, University of California, Davis, One Shields Ave, Davis, CA 95616, USA

## Abstract

The title compound, [Co^II^(C_2_H_8_N_2_)_3_]Cl_2_, was obtained unexpectedly as the product of an attempted solvothermal synthesis of cobalt selenide from the elements in the presence of NH_4_Cl in ethyl­enedi­amine solvent. The three chelate rings of the distorted octa­hedral [Co(C_2_H_8_N_2_)_3_]^2+^ complex cation adopt twisted conformations about their C—C bonds. The spread of *cis*-N—Co—N bond angles [80.17 (6)–98.10 (6)°] in the title compound is considerably greater than the equivalent data for [Co^III^(C_2_H_8_N_2_)_3_]Cl_3_ [Takamizawa *et al.* (2008 ▶). *Angew. Chem. Int. Ed.* **47**, 1689–1692]. In the crystal, the components are linked by numerous N—H⋯Cl hydrogen bonds, generating a three-dimensional network in which the cationic complexes are stacked in columns along [010] and separated by columns of chloride anions.

## Related literature

The corresponding Co^III^–tris-ethyl­enedi­amine complex with chloride counter-anions has been reported by Takamizawa *et al.* (2008 ▶).
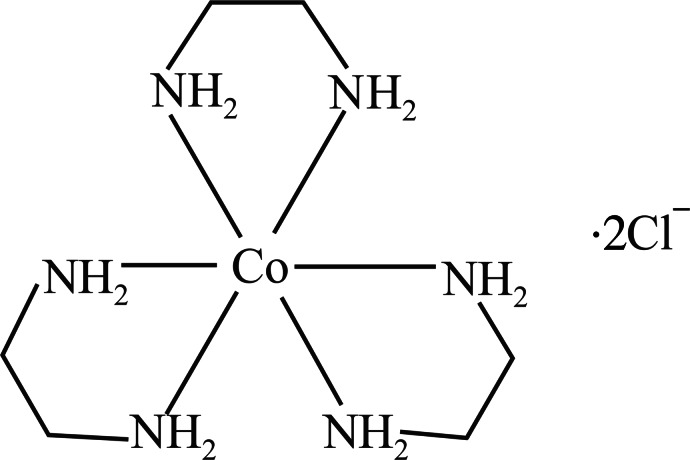


## Experimental

### 

#### Crystal data

[Co(C_2_H_8_N_2_)_3_]Cl_2_
*M* *_r_* = 310.14
Orthorhombic, 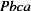

*a* = 8.1590 (8) Å
*b* = 17.047 (3) Å
*c* = 20.3974 (14) Å
*V* = 2837.0 (6) Å^3^
*Z* = 8
Cu *K*α radiation
μ = 12.81 mm^−1^
*T* = 90 K
0.31 × 0.17 × 0.15 mm

#### Data collection

Bruker APEXII CCD diffractometer
Absorption correction: multi-scan (*SADABS*; Bruker, 2003 ▶) *T*_min_ = 0.109, *T*_max_ = 0.243
17930 measured reflections
2700 independent reflections
2437 reflections with *I* > 2σ(*I*)
*R*_int_ = 0.042

#### Refinement

*R*[*F* ^2^ > 2σ(*F* ^2^)] = 0.024
*wR*(*F* ^2^) = 0.059
*S* = 1.06
2700 reflections
232 parameters
All H-atom parameters refined
Δρ_max_ = 0.26 e Å^−3^
Δρ_min_ = −0.37 e Å^−3^

### 

Data collection: *APEX2* (Bruker, 2003 ▶); cell refinement: *SAINT* (Bruker, 2003 ▶); data reduction: *SAINT*; program(s) used to solve structure: *SHELXS97* (Sheldrick, 2008 ▶); program(s) used to refine structure: *SHELXL97* (Sheldrick, 2008 ▶); molecular graphics: *Mercury* (Macrae *et al.*, 2008 ▶); software used to prepare material for publication: *SHELXL97*.

## Supplementary Material

Crystal structure: contains datablock(s) I, global. DOI: 10.1107/S1600536813013135/hb7079sup1.cif

Structure factors: contains datablock(s) I. DOI: 10.1107/S1600536813013135/hb7079Isup2.hkl

Additional supplementary materials:  crystallographic information; 3D view; checkCIF report

## Figures and Tables

**Table 1:** Selected bond lengths (Å)

Co—N1	2.1540 (15)
Co—N3	2.1558 (15)
Co—N2	2.1635 (15)
Co—N5	2.1748 (15)
Co—N4	2.1767 (15)
Co—N6	2.1791 (16)

**Table 2:** Hydrogen-bond geometry (Å, °)

*D*—H⋯*A*	*D*—H	H⋯*A*	*D*⋯*A*	*D*—H⋯*A*
N1—H1*B*⋯Cl2	0.82 (2)	2.48 (3)	3.2839 (17)	167 (2)
N1—H1*A*⋯Cl2^i^	0.86 (2)	2.57 (2)	3.3056 (16)	145.4 (18)
N2—H2*A*⋯Cl1^ii^	0.83 (2)	2.92 (2)	3.5494 (16)	133.6 (17)
N2—H2*A*⋯Cl2^iii^	0.83 (2)	2.94 (2)	3.5887 (17)	135.8 (17)
N2—H2*B*⋯Cl1	0.88 (2)	2.65 (2)	3.4566 (17)	152.5 (18)
N5—H5*B*⋯Cl1^ii^	0.87 (2)	2.51 (2)	3.3770 (16)	173.1 (19)
N5—H5*A*⋯Cl1^iv^	0.82 (2)	2.70 (2)	3.4514 (18)	152.4 (19)
N6—H6*B*⋯Cl1	0.88 (3)	2.66 (3)	3.4552 (18)	150.3 (19)
N6—H6*A*⋯Cl1^v^	0.83 (2)	2.71 (2)	3.4653 (16)	152.9 (19)
N3—H3*A*⋯Cl1^iv^	0.88 (2)	2.59 (2)	3.4075 (17)	154.2 (17)
N3—H3*B*⋯Cl2^i^	0.82 (2)	2.49 (2)	3.2560 (16)	156 (2)
N4—H4*A*⋯Cl2	0.85 (2)	2.68 (2)	3.5003 (17)	161 (2)
N4—H4*B*⋯Cl2^iii^	0.87 (2)	2.55 (2)	3.2919 (16)	143.5 (19)

Symmetry codes: (i) 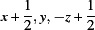; (ii) 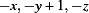; (iii) 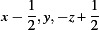; (iv) 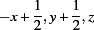; (v) 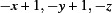.

**Fig. 1.**
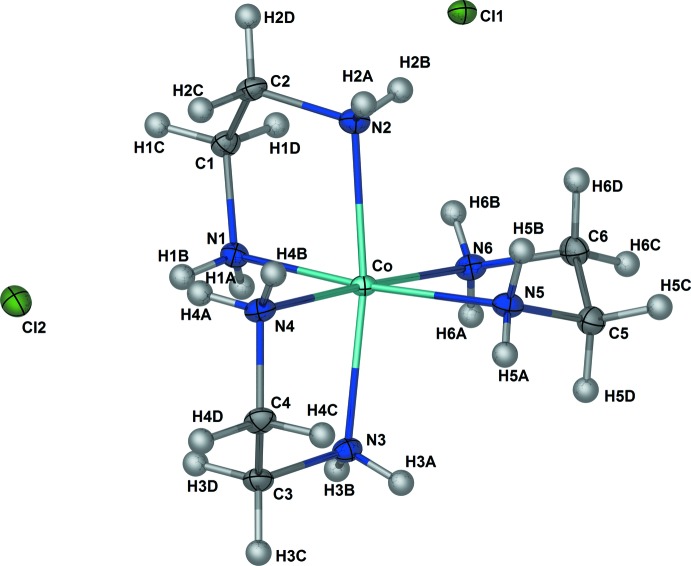
The molecular structure of (I) with 50% probability displacement ellipsoids for non-H atoms.

**Fig. 2.**
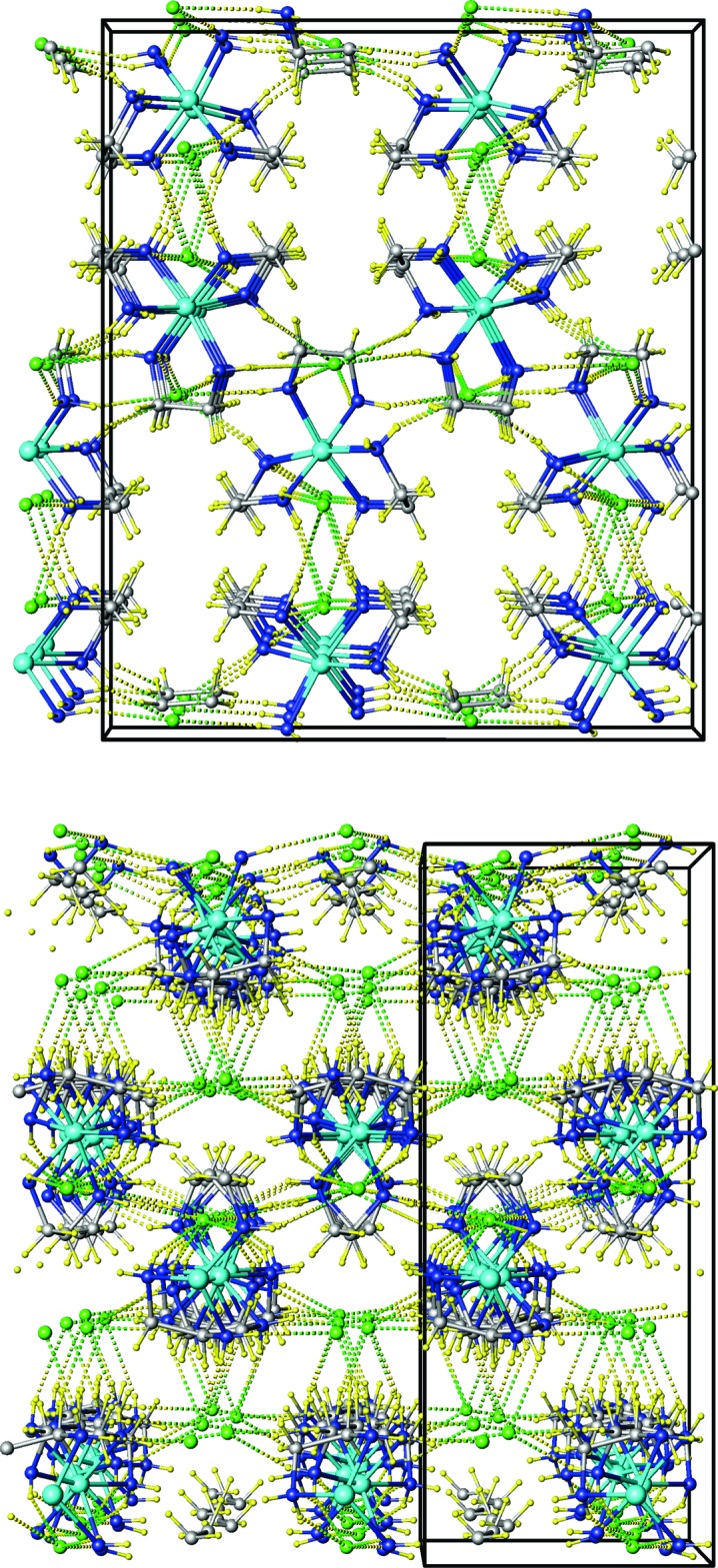
Packing of the molecules in the crystal structure of (I) along (top) [100] and (bottom) [010] crystallographic directions. Cl–H distances in the range from 2.45 to 2.70 Å are shown with dashed lines.

## supplementary crystallographic information

### Comment

In the chiral Co(II)(en)_3_ cationic complex (Fig. 1) N atoms form distorted octahedron around central Co atom, *cis* angles deviate from 90° by less than 10°. Both Λ and Δ isomers are present in equal amounts in the centrosymmetric crystal structure.

Co(en)_3_ cationic complexes are stacked in columns along [010] direction (Figure 2) and separated by the columns of Cl anions. There two types of chlorine anions in the crystal structure. Cl1 has distorted octahedral coordination by 6 hydrogen atoms from 4 different Co(en)_3_ complexes. Cl2 has distorted trigonal bipyramid coordination by 5 hydrogen atoms from 2 different Co(en)_3_ complexes. H···Cl distances vary from 2.45 to 2.70 Å.

The corresponding Co(III) trisethylenediamine complex with chloride counter-anions has been reported. (Takamizawa *et al.*, 2008). The Co(III)(en)_3_ cationic complex is more regular: *cis* angles deviate from 90° by less than 4°.

### Experimental

The title compound, [Co(C_2_N_2_H_8_)_3_]Cl_2_, was obtained unintentionally as the product of an attempted synthesis of cobalt selenide. Co (64 mg), Se (86 mg), and NH_4_Cl (100 mg) were reacted in ethylenediamine (en) solvent (30 ml). Reaction was performed in closed hydrothermal vessel at 180°C for 48 h. Degree of the vessel filling was 70%. Irregular moisture-sensitive yellow crystals of the title compound were recovered.

### Refinement

H atoms bonded to N and C atoms were located in a difference Fourier maps and refined without any restraints.

### Crystal data

**Table:**

[Co(C_2_H_8_N_2_)_3_]Cl_2_	*D*_x_ = 1.451 Mg m^−^^3^
*M**_r_* = 310.14	Melting point: not measured K
Orthorhombic, *P**b**c**a*	Cu *K*α radiation, λ = 1.54178 Å
*a* = 8.1590 (8) Å	Cell parameters from 6618 reflections
*b* = 17.047 (3) Å	θ = 4.3–71.0°
*c* = 20.3974 (14) Å	µ = 12.81 mm^−^^1^
*V* = 2837.0 (6) Å^3^	*T* = 90 K
*Z* = 8	Irregular, yellow
*F*(000) = 1304	0.31 × 0.17 × 0.15 mm

### Data collection

**Table:**

Bruker APEXII CCD diffractometer	2700 independent reflections
Radiation source: fine-focus sealed tube	2437 reflections with *I* > 2σ(*I*)
Graphite monochromator	*R*_int_ = 0.042
φ and ω scans	θ_max_ = 72.0°, θ_min_ = 4.3°
Absorption correction: multi-scan (*SADABS*; Bruker, 2003)	*h* = −9→9
*T*_min_ = 0.109, *T*_max_ = 0.243	*k* = −19→20
17930 measured reflections	*l* = −24→18

### Refinement

**Table:**

Refinement on *F*^2^	Primary atom site location: structure-invariant direct methods
Least-squares matrix: full	Secondary atom site location: difference Fourier map
*R*[*F*^2^ > 2σ(*F*^2^)] = 0.024	Hydrogen site location: inferred from neighbouring sites
*wR*(*F*^2^) = 0.059	All H-atom parameters refined
*S* = 1.06	*w* = 1/[σ^2^(*F*_o_^2^) + (0.0273*P*)^2^ + 1.3198*P*] where *P* = (*F*_o_^2^ + 2*F*_c_^2^)/3
2700 reflections	(Δ/σ)_max_ = 0.001
232 parameters	Δρ_max_ = 0.26 e Å^−^^3^
0 restraints	Δρ_min_ = −0.37 e Å^−^^3^

### Special details

**Table:**

Geometry. All e.s.d.'s (except the e.s.d. in the dihedral angle between two l.s. planes) are estimated using the full covariance matrix. The cell e.s.d.'s are taken into account individually in the estimation of e.s.d.'s in distances, angles and torsion angles; correlations between e.s.d.'s in cell parameters are only used when they are defined by crystal symmetry. An approximate (isotropic) treatment of cell e.s.d.'s is used for estimating e.s.d.'s involving l.s. planes.
Refinement. Refinement of *F*^2^ against ALL reflections. The weighted *R*-factor *wR* and goodness of fit *S* are based on *F*^2^, conventional *R*-factors *R* are based on *F*, with *F* set to zero for negative *F*^2^. The threshold expression of *F*^2^ > σ(*F*^2^) is used only for calculating *R*-factors(gt) *etc*. and is not relevant to the choice of reflections for refinement. *R*-factors based on *F*^2^ are statistically about twice as large as those based on *F*, and *R*- factors based on ALL data will be even larger.

### Fractional atomic coordinates and isotropic or equivalent isotropic displacement parameters (Å^2^)

**Table:**

	*x*	*y*	*z*	*U*_iso_*/*U*_eq_	
Co	0.22206 (3)	0.636475 (16)	0.101937 (14)	0.01130 (9)	
Cl1	0.22916 (5)	0.38557 (2)	0.02211 (2)	0.01517 (10)	
Cl2	0.19514 (5)	0.63342 (2)	0.32464 (2)	0.01878 (11)	
N1	0.31525 (19)	0.56644 (9)	0.18154 (8)	0.0144 (3)	
H1A	0.419 (3)	0.5613 (12)	0.1780 (10)	0.021 (6)*	
H1B	0.297 (3)	0.5888 (15)	0.2164 (12)	0.023 (6)*	
N2	0.04364 (19)	0.54264 (9)	0.09943 (8)	0.0155 (3)	
H2A	−0.051 (3)	0.5597 (13)	0.0934 (10)	0.019 (6)*	
H2B	0.068 (3)	0.5088 (13)	0.0683 (11)	0.023 (6)*	
C1	0.2324 (2)	0.48967 (10)	0.18119 (9)	0.0169 (4)	
H1C	0.238 (2)	0.4648 (12)	0.2236 (10)	0.014 (5)*	
H1D	0.285 (2)	0.4581 (12)	0.1472 (10)	0.016 (5)*	
C2	0.0533 (2)	0.50142 (11)	0.16281 (9)	0.0167 (4)	
H2C	0.003 (2)	0.5343 (12)	0.1971 (10)	0.020 (5)*	
H2D	0.001 (2)	0.4511 (12)	0.1614 (10)	0.018 (5)*	
N6	0.37572 (19)	0.57543 (9)	0.03100 (8)	0.0156 (3)	
H5B	0.044 (3)	0.6765 (13)	0.0010 (10)	0.023 (6)*	
H5A	0.135 (3)	0.7409 (15)	0.0172 (10)	0.024 (6)*	
N5	0.14074 (19)	0.69328 (9)	0.01218 (8)	0.0152 (3)	
H6A	0.474 (3)	0.5868 (13)	0.0328 (10)	0.020 (5)*	
H6B	0.372 (3)	0.5247 (15)	0.0387 (11)	0.029 (6)*	
C5	0.2573 (2)	0.67473 (11)	−0.04124 (9)	0.0179 (4)	
H5D	0.351 (2)	0.7073 (12)	−0.0344 (9)	0.016 (5)*	
H5C	0.211 (2)	0.6829 (13)	−0.0850 (11)	0.021 (5)*	
C6	0.3113 (2)	0.59006 (11)	−0.03548 (9)	0.0182 (4)	
H6D	0.217 (2)	0.5559 (11)	−0.0402 (9)	0.008 (4)*	
H6C	0.391 (3)	0.5790 (12)	−0.0690 (10)	0.019 (5)*	
N3	0.38406 (18)	0.73377 (9)	0.12091 (8)	0.0146 (3)	
H3A	0.385 (2)	0.7681 (13)	0.0886 (10)	0.016 (5)*	
H3B	0.478 (3)	0.7190 (13)	0.1278 (11)	0.023 (6)*	
N4	0.07178 (18)	0.70245 (9)	0.17074 (8)	0.0155 (3)	
H4B	−0.032 (3)	0.7045 (13)	0.1610 (10)	0.027 (6)*	
H4A	0.079 (3)	0.6796 (14)	0.2080 (11)	0.026 (6)*	
C3	0.3253 (2)	0.77480 (11)	0.17991 (9)	0.0175 (4)	
H3D	0.353 (2)	0.7452 (12)	0.2186 (9)	0.011 (5)*	
H3C	0.374 (2)	0.8260 (12)	0.1837 (9)	0.016 (5)*	
C4	0.1402 (2)	0.78212 (10)	0.17682 (10)	0.0178 (4)	
H4C	0.109 (2)	0.8107 (12)	0.1386 (10)	0.018 (5)*	
H4D	0.097 (3)	0.8110 (12)	0.2159 (10)	0.020 (5)*	

### Atomic displacement parameters (Å^2^)

**Table:**

	*U*^11^	*U*^22^	*U*^33^	*U*^12^	*U*^13^	*U*^23^
Co	0.00953 (15)	0.00972 (15)	0.01465 (16)	−0.00031 (10)	−0.00038 (10)	0.00023 (10)
Cl1	0.0154 (2)	0.0123 (2)	0.0178 (2)	−0.00106 (14)	−0.00053 (15)	0.00024 (15)
Cl2	0.0122 (2)	0.0245 (2)	0.0197 (2)	0.00031 (15)	0.00082 (16)	−0.00254 (17)
N1	0.0125 (8)	0.0140 (7)	0.0166 (8)	0.0004 (6)	−0.0007 (6)	−0.0010 (6)
N2	0.0127 (8)	0.0134 (8)	0.0204 (9)	0.0004 (6)	−0.0011 (6)	−0.0001 (6)
C1	0.0196 (9)	0.0125 (9)	0.0186 (9)	−0.0005 (7)	−0.0005 (7)	0.0022 (7)
C2	0.0176 (9)	0.0119 (8)	0.0207 (10)	−0.0024 (7)	0.0022 (7)	0.0013 (7)
N6	0.0120 (8)	0.0138 (8)	0.0211 (8)	0.0009 (6)	0.0007 (6)	0.0007 (6)
N5	0.0133 (7)	0.0118 (8)	0.0204 (8)	0.0009 (6)	−0.0020 (6)	−0.0005 (6)
C5	0.0184 (9)	0.0175 (9)	0.0177 (10)	−0.0011 (7)	−0.0012 (7)	0.0010 (7)
C6	0.0189 (9)	0.0177 (9)	0.0181 (10)	0.0006 (7)	0.0022 (8)	−0.0030 (7)
N3	0.0114 (7)	0.0136 (7)	0.0189 (8)	−0.0005 (6)	−0.0006 (6)	0.0020 (6)
N4	0.0132 (8)	0.0129 (7)	0.0205 (9)	−0.0011 (6)	0.0019 (6)	0.0000 (6)
C3	0.0201 (9)	0.0125 (9)	0.0198 (10)	−0.0034 (7)	−0.0015 (7)	0.0003 (7)
C4	0.0210 (9)	0.0106 (8)	0.0217 (10)	0.0001 (7)	0.0023 (8)	−0.0001 (7)

### Geometric parameters (Å, º)

**Table:**

Co—N1	2.1540 (15)	N5—C5	1.480 (2)
Co—N3	2.1558 (15)	N5—H5B	0.87 (2)
Co—N2	2.1635 (15)	N5—H5A	0.82 (2)
Co—N5	2.1748 (15)	C5—C6	1.514 (3)
Co—N4	2.1767 (15)	C5—H5D	0.95 (2)
Co—N6	2.1791 (16)	C5—H5C	0.98 (2)
N1—C1	1.473 (2)	C6—H6D	0.967 (19)
N1—H1A	0.86 (2)	C6—H6C	0.96 (2)
N1—H1B	0.82 (2)	N3—C3	1.472 (2)
N2—C2	1.473 (2)	N3—H3A	0.88 (2)
N2—H2A	0.83 (2)	N3—H3B	0.82 (2)
N2—H2B	0.88 (2)	N4—C4	1.474 (2)
C1—C2	1.522 (2)	N4—H4B	0.87 (2)
C1—H1C	0.96 (2)	N4—H4A	0.85 (2)
C1—H1D	0.98 (2)	C3—C4	1.517 (2)
C2—H2C	0.99 (2)	C3—H3D	0.963 (19)
C2—H2D	0.96 (2)	C3—H3C	0.96 (2)
N6—C6	1.475 (2)	C4—H4C	0.95 (2)
N6—H6A	0.83 (2)	C4—H4D	1.00 (2)
N6—H6B	0.88 (3)		
			
N1—Co—N3	94.28 (6)	H6A—N6—H6B	105 (2)
N1—Co—N2	81.11 (6)	C5—N5—Co	109.18 (11)
N3—Co—N2	170.27 (6)	C5—N5—H5B	108.7 (14)
N1—Co—N5	171.51 (6)	Co—N5—H5B	110.5 (14)
N3—Co—N5	89.75 (6)	C5—N5—H5A	109.8 (15)
N2—Co—N5	95.97 (6)	Co—N5—H5A	110.6 (15)
N1—Co—N4	89.95 (6)	H5B—N5—H5A	108 (2)
N3—Co—N4	80.33 (6)	N5—C5—C6	109.49 (15)
N2—Co—N4	91.05 (6)	N5—C5—H5D	106.3 (12)
N5—Co—N4	98.10 (6)	C6—C5—H5D	108.2 (12)
N1—Co—N6	91.88 (6)	N5—C5—H5C	113.1 (12)
N3—Co—N6	97.69 (6)	C6—C5—H5C	108.5 (13)
N2—Co—N6	91.05 (6)	H5D—C5—H5C	111.1 (17)
N5—Co—N6	80.17 (6)	N6—C6—C5	109.64 (15)
N4—Co—N6	177.41 (6)	N6—C6—H6D	105.8 (11)
C1—N1—Co	109.07 (11)	C5—C6—H6D	109.6 (11)
C1—N1—H1A	111.3 (14)	N6—C6—H6C	112.2 (12)
Co—N1—H1A	110.0 (14)	C5—C6—H6C	109.1 (13)
C1—N1—H1B	109.6 (16)	H6D—C6—H6C	110.4 (16)
Co—N1—H1B	109.5 (16)	C3—N3—Co	108.21 (11)
H1A—N1—H1B	107 (2)	C3—N3—H3A	107.4 (13)
C2—N2—Co	107.21 (11)	Co—N3—H3A	112.6 (13)
C2—N2—H2A	110.2 (14)	C3—N3—H3B	108.1 (16)
Co—N2—H2A	111.6 (15)	Co—N3—H3B	111.7 (15)
C2—N2—H2B	108.0 (14)	H3A—N3—H3B	109 (2)
Co—N2—H2B	110.4 (14)	C4—N4—Co	108.49 (11)
H2A—N2—H2B	109 (2)	C4—N4—H4B	110.5 (15)
N1—C1—C2	108.96 (14)	Co—N4—H4B	114.9 (15)
N1—C1—H1C	111.3 (12)	C4—N4—H4A	108.7 (16)
C2—C1—H1C	109.1 (12)	Co—N4—H4A	107.5 (15)
N1—C1—H1D	106.9 (12)	H4B—N4—H4A	107 (2)
C2—C1—H1D	108.7 (11)	N3—C3—C4	109.24 (15)
H1C—C1—H1D	111.9 (16)	N3—C3—H3D	110.2 (11)
N2—C2—C1	109.29 (15)	C4—C3—H3D	108.0 (11)
N2—C2—H2C	109.2 (12)	N3—C3—H3C	111.2 (12)
C1—C2—H2C	107.6 (12)	C4—C3—H3C	110.0 (12)
N2—C2—H2D	112.1 (12)	H3D—C3—H3C	108.2 (16)
C1—C2—H2D	108.4 (12)	N4—C4—C3	107.74 (14)
H2C—C2—H2D	110.2 (17)	N4—C4—H4C	107.6 (12)
C6—N6—Co	108.95 (11)	C3—C4—H4C	109.8 (12)
C6—N6—H6A	110.3 (15)	N4—C4—H4D	112.8 (12)
Co—N6—H6A	114.7 (15)	C3—C4—H4D	110.8 (12)
C6—N6—H6B	108.5 (14)	H4C—C4—H4D	108.0 (16)
Co—N6—H6B	109.3 (14)		

### Hydrogen-bond geometry (Å, º)

**Table:**

*D*—H···*A*	*D*—H	H···*A*	*D*···*A*	*D*—H···*A*
N1—H1*B*···Cl2	0.82 (2)	2.48 (3)	3.2839 (17)	167 (2)
N1—H1*A*···Cl2^i^	0.86 (2)	2.57 (2)	3.3056 (16)	145.4 (18)
N2—H2*A*···Cl1^ii^	0.83 (2)	2.92 (2)	3.5494 (16)	133.6 (17)
N2—H2*A*···Cl2^iii^	0.83 (2)	2.94 (2)	3.5887 (17)	135.8 (17)
N2—H2*B*···Cl1	0.88 (2)	2.65 (2)	3.4566 (17)	152.5 (18)
N5—H5*B*···Cl1^ii^	0.87 (2)	2.51 (2)	3.3770 (16)	173.1 (19)
N5—H5*A*···Cl1^iv^	0.82 (2)	2.70 (2)	3.4514 (18)	152.4 (19)
N6—H6*B*···Cl1	0.88 (3)	2.66 (3)	3.4552 (18)	150.3 (19)
N6—H6*A*···Cl1^v^	0.83 (2)	2.71 (2)	3.4653 (16)	152.9 (19)
N3—H3*A*···Cl1^iv^	0.88 (2)	2.59 (2)	3.4075 (17)	154.2 (17)
N3—H3*B*···Cl2^i^	0.82 (2)	2.49 (2)	3.2560 (16)	156 (2)
N4—H4*A*···Cl2	0.85 (2)	2.68 (2)	3.5003 (17)	161 (2)
N4—H4*B*···Cl2^iii^	0.87 (2)	2.55 (2)	3.2919 (16)	143.5 (19)

Symmetry codes: (i) *x*+1/2, *y*, −*z*+1/2; (ii) −*x*, −*y*+1, −*z*; (iii) *x*−1/2, *y*, −*z*+1/2; (iv) −*x*+1/2, *y*+1/2, *z*; (v) −*x*+1, −*y*+1, −*z*.
